# Safety evaluation and application of lactic acid bacteria and yeast strains isolated from Sichuan broad bean paste

**DOI:** 10.1002/fsn3.3129

**Published:** 2022-12-19

**Authors:** Lu Feng, Jinhong Gu, Linjie Guo, Guangqing Mu, Yanfeng Tuo

**Affiliations:** ^1^ School of Food Science and Technology Dalian Polytechnic University Dalian China

**Keywords:** broad bean paste, fermentation, lactic acid bacteria, safety evaluation, yeast

## Abstract

Broad bean paste is one of the most popular characteristic traditional fermented bean products in China, which is prepared by mixed fermentation of a variety of microorganisms, among which lactic acid bacteria and yeast played an important role in the improvement of the fermented broad bean paste quality. However, the traditional open‐air fermentation of broad bean paste brought some risks of harmful microorganisms. In this study, the safety and fermentation ability of lactic acid bacteria and yeast strains isolated from traditional broad bean paste was evaluated. The results showed that the protease activity of the strain *Lactobacillus plantarum* DPUL‐J5 (366.73 ± 9.00 U/L) and *Pichia kudriavzevii* DPUY‐J5 (237.18 ± 10.93 U/L) were the highest. Both strains produced little biogenic amines, and did not exhibit α‐hemolytic activity or antibiotic resistance for some of the antibiotics most used in human medicine. Furthermore, the broad bean paste fermentation involving DPUL‐J5 and DPUY‐J5 was beneficial for accumulating higher total acid (1.69 ± 0.01 g/100 g), amino‐acid nitrogen (0.85 ± 0.03 g/100 g), and more volatile flavor compounds, meanwhile, reducing the levels of biogenic amines and aflatoxin B1. Therefore, this study provided a new strategy to improve the safety and quality of traditional broad bean paste.

## Graphical abstract

Broad bean paste is one of the main traditional fermented products in China, and the microorganisms involving in the fermentation process are susceptible to the production environment. Lactic acid bacteria and yeast play an important role in the development of the fermented bean paste property.

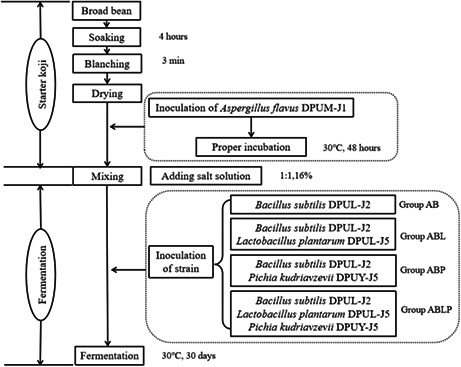

## 1 INTRODUCTION

Broad bean paste is a famous Chinese condiment made by fermenting broad bean (*Vicia faba L*.) with microorganisms, some of it has red pepper (*Capsicum annuum L*.), which is very popular in Sichuan and Chongqing provinces, which develops a unique flavor due to the long‐term fermentation by mold, yeast, and bacteria (Li et al., 2018; Zhang et al., 2020). The *Aspergillus* and *Bacillus* were necessary for koji preparation for broad bean paste production, which can secrete protease, amylase, and other enzymes to decompose proteins, starch, and other macromolecular substances in raw materials, produce large amounts of small molecules, such as amino acids and sugars in broad bean paste, which can provide carbon and nitrogen sources for the growth of other microorganisms, and improve the quality of fermented products (Li et al., 2021; Ruiz‐Capillas & Herrero, 2019). Lactic acid bacteria (LAB) and yeast, as the predominant microorganisms in the post‐fermentation process of broad bean paste, had a considerable impact on the development of the nutrition composition and flavor compounds, such as free amino acids, organic acids, and volatile aroma substances. The proteases secreted by LAB and yeast were generally abundant, but other enzymes were too little.

Traditional broad bean paste was fermented in the open air; there may be some safety problems such as harmful microorganism in the fermentation process. At the same time, some microorganisms in the broad bean paste can produce harmful substances such as aflatoxins B1 (AFB1) and biogenic amines (BAs), which may pose a threat to human health. Therefore, it is very necessary to evaluate and screen safe starter cultures to improve the safety and quality of the fermentation product. AFB1 is well recognized as one of the most critical mycotoxins and human carcinogens, which was produced by a genus of *Aspergillus* and commonly observed in numerous crops and fermented food (Zhang et al., 2020). BAs are organic nitrogenous compounds produced by microorganisms in food through the decarboxylation of an amino acid (Ruiz‐Capillas & Herrero, 2019; Saaid et al., 2009). The formation of biogenic amines has two conditions, one was free amino acids and another was amine‐producing microorganisms (Linares et al., 2012), which were especially rich in fermented bean products.

Lactic acid bacteria were reported to be strong BA producers in different fermented foods, and yeast produced fewer amines (Spano et al., 2010). Some molds also can produce biogenic amines, and *Aspergillus oryzae* and *Mucor* isolated from traditional Sichuan bean paste can produce tyramine (Hao & Sun, 2020). Although *Bacillus* can be used as a starter culture, some species can also produce biogenic amines, and their safety needs to be evaluated (Jae‐Hyung et al., 2019). In addition to producing BAs, the starters have potential risks of having antibiotic resistance and hemolysis. It is imperative to screen microorganisms with no antibiotic resistance before using them as starters (Imperial & Ibana, 2016). Moreover, strains used in food production were required not to show hemolytic activity (Marty et al., 2012).

In this study, the safety and fermentation ability of lactic acid bacteria and yeast isolated from traditional broad bean paste were evaluated, and they were applied to the fermentation of broad bean paste, which provides a new strategy for improving the quality of broad bean paste and reducing risks.

## 2 MATERIALS AND METHODS

### 2.1 Strains and cultural conditions

In this study, LAB and yeast strains were isolated from traditional broad bean paste from the Pidu district (Chengdu city, Sichuan Province, China). There were 16 strains of LAB, which were cultured on Man, Rogosa, and Sharpe (MRS) medium for 18 h, and there were 14 yeasts which were cultured on yeast extract peptone dextrose (YPD) medium for 18 h. *Aspergillus flavus* DPUM‐J1 and *Bacillus subtilis* DPUL‐J2 were also isolated from traditional broad bean paste and did not produce AFB1 or BAs. *Aspergillus* and *Bacillus* had high protease and amylase activities and have been used as starters in fermented soybean paste (Li et al., 2021). All strains were preserved in the Dalian probiotics function research key laboratory, Dalian Polytechnic University laboratory.

### 2.2 The determination of the biogenic amines‐producing ability of the lactic acid bacteria and yeast strains

The production of BAs by LAB and yeast strains was determined according to the reported methods (Li et al., 2021). The LAB and yeast strains were inoculated in the modified MRS or YPD broth which contained seven 0.1% (w/v) BA precursors, including L‐tyrosine, L‐histidine hydrochloride, L‐lysine hydrochloride, L‐tryptophan hydrochloride, L‐arginine hydrochloride, L‐ornithine hydrochloride, L‐phenylalanine hydrochloride (Beijing Solarbio Science & Technology), and 0.005% (w/v) pyridoxal‐5‐phosphate‐HCl (Sigma‐Aldrich). The LAB were cultured at 37°C and the yeast at 28°C for 48 h in the modified MRS or YPD broth, respectively. The 750 μl of each culture suspension was mixed with 750 μl dansyl chloride (Sigma‐Aldrich) and 150 μl saturated sodium bicarbonate. The mixture was incubated for 30 min at 40°C. Residual dansyl chloride was removed by adding 150 μl ammonium hydroxide and incubated at 40°C for 15 min, complemented to 1.5 ml with acetonitrile through a 0.22 μm syringe filter, and analyzed by HPLC equipped with a Zorbax Eclipse XDB‐C18 column (4.6 mm × 250 mm, 5 μm) at 254 nm. The injection volume for the HPLC system was 30 μl. The flow rate was 1.0 ml/min, and the temperature was set at 40°C.

### 2.3 The determination of protease activities of lactic acid bacteria and yeast strains

After previous laboratory studies, amylase and lipase activities of lactic acid bacteria and yeast were low, so only protease activity was measured. Five grams of broad bean and 50 ml of 2.5 mol/L NaCl solution were added into a conical flask and sterilized at 121°C for 20 min to prepare the fermentation medium. LAB and yeast were 2% inoculated into the fermentation medium and cultured at 37°C and 28°C for 48 h, respectively. After cultivation, the culture of LAB and yeast were centrifuged at 8000 × *g* (7630 rpm in a FA‐45‐6‐30 rotor, centrifuge 5804R, Eppendorf AG) in 4°C for 10 min to obtain supernatant as a crude enzyme solution for determining protease activity by using Total Protease (Protease) ELISA Kit (BIO).

### 2.4 Identification

The strain with high protease activity and extremely low BAs production was identified by 16S rDNA and 26S rRNA gene sequences analysis. The gene sequences were compared on NCBI using BLAST (http://blast.ncbi.nlm.nih.gov/Blast.cgi) for homology comparison, the recognized standard sequence data were obtained from the Gen Bank database, and the MAGE (v. 7.0, https://www.megasoftware.net/) was used to make a phylogenetic tree.

### 2.5 Hemolytic activity test

Hemolysis activity was determined according to the method described by Jeong et al. (2014). The LAB and yeast were streaked on the Columbia agar medium (Oxoid Ltd) containing 5% (v/v) sheep blood (Beijing Solarbio Science & Technology Co., Ltd) and cultured at 37°C and 30°C for 48 h to detect patterns of hemolysis. The appearance of a grass‐green ring indicated ɑ‐hemolytic activity; the appearance of a well‐defined and transparent hemolytic ring indicated β‐hemolytic activity, and no change was no hemolysis. *Listeria monocytogenes* SHBCC D15669 (Shanghai Bioresource Collection Center) was used as a positive control for hemolytic analyses. Three independent experiments were conducted.

### 2.6 Antibiotic susceptibility test

The antibiotic resistance profiles of the LAB and yeast were conducted by the method described by Nami et al. (2014). Ten antibiotics (Shanghai yuanye Bio‐Technology Co., Ltd), including ampicillin, chloramphenicol, ciprofloxacin, kanamycin, gentamicin, penicillin, tetracycline, vancomycin, clindamycin, and levofloxacin, were selected for sensitivity test of LAB strain. Identification of antibiotic susceptibility by inhibition zone diameter: susceptible >20 mm, intermediate resistant 15–19 mm, and resistant ≤14 mm (Clinical and Laboratory Standards Institute, 2012). Seven antibiotics including econazole, itraconazole, clotrimazole, ketoconazole, miconazole, amphotericin, and fluconazole were selected for yeast antibiotic sensitivity test. Identification of yeast antibiotic susceptibility by inhibition zone diameter: susceptible≥20 mm, intermediate resistant 10–20 mm, and resistant ≤10 mm (Clinical and Laboratory Standards Institute, 2012).

### 2.7 Broad bean paste preparation

**FIGURE 1:**
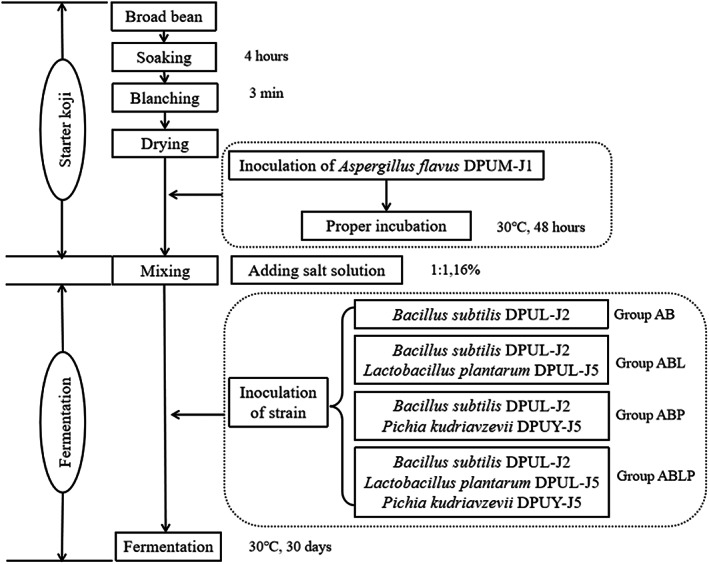
The procedures to prepare broad bean paste. (AB) Inoculated with *Bacillus subtilis* DPUL‐J2 in broad bean koji. (ABL) Inoculated with *Bacillus subtilis* DPUL‐J2 and *Lactobacillus plantarum* DPUL‐J5 in broad bean koji. (ABP) Inoculated with *Bacillus subtilis* DPUL‐J2 and *Pichia kudriavzevii* in broad bean koji. (ABLP) Inoculated with *Bacillus subtilis* DPUL‐J2, *lactobacillus plantarum* DPUL‐J5, and *Pichia kudriavzevii* in broad bean koji. *Aspergillus flavus* DPUM‐J1 was used to prepare koji. The material‐to‐liquid ratio was 1:1 (w/v) and submerged into a brine solution (16% w/v, NaCl). The suspension concentration of strain was 10^8^–10^9^ CFU/ml, and the inoculation amount was 2% (v/v)

The fermentation process of broad bean paste mainly included three stages: preparation of seed koji, preparation of broad bean koji, and mixed fermentation with brine (Zhu et al., 2017). Firstly, *Aspergillus flavus* DPUM‐J1 was inoculated in potato dextrose agar (PDA) medium and cultured at 28°C for 72 h to obtain spores as seed koji. Subsequently, the shelled broad beans were soaked, blanched, dried, mixed with the flour evenly at the ratio of 1:0.3 (w/w), and then the mixture was inoculated with *Aspergillus flavus* DPUM‐J1 spore suspension (inoculation amount for 1.5%, v/w), cultivated at 30°C for 48 h as koji. The koji was turned once a day in order to prevent the koji from clumping until the surface of the broad bean was coated with *A. flavus* DPUM‐J1 mycelium. Finally, the prepared broad bean koji was inoculated with 2% *Bacillus subtilis* DPUL‐J2 and divided into four groups according to Figure 1, mixed with brine in a 1:1 ratio (the salt content of 16%, v/v), 2% inoculated with different strains for fermentation, covered the bottle with gauze, and fermented for 30 days at 30°C. During the fermentation process, stirred for every 5 days. Samples of the fermented broad bean pastes were taken on the 0, 5, 10, 15, 20, 25, and 30 days for chemical properties analysis.

### 2.8 Chemical properties analysis of broad bean paste

The pH level of the broad bean paste samples was determined by pH meter. Additionally, the amino acid nitrogen (AAN) and total acidity (TA) contents of the broad bean paste were analyzed according to the National Standards. The reducing sugar content was determined by the 3,5‐dinitrosalicylic acid (DNS) method (Wu et al., 2018).

### 2.9 The determination of biogenic amines and aflatoxin B1 content in broad bean paste

Biogenic amines content in broad bean paste samples was detected in accordance with the methods described by Liu et al. (2021) and Kim and Ji (2015) with a slight modification. Five grams of broad bean paste sample was accurately weighed from different fermentation periods, and 20 ml of 5% trichloroacetic acid solution was added to remove protein; the mixture was homogenized using an orbital mixer for 60 min and centrifuged at 8000 *× g* (7630 rpm) at 4°C for 10 min to collected the supernatant, and the residues were mixed with 5% trichloroacetic acid and centrifuged again. The supernatant was merged twice and adjusted to 50 ml with 5% trichloroacetic acid, took 10 ml of the above extract and added an equal volume of n‐hexane (Guangdong Guanghua Sci‐Tech Co., Ltd) to remove lipid, vortexed for 5 min, and discarded the upper organic phase. 750 μl of the extract was transferred to a tube， added 150 μl of saturated sodium carbonate solution and 750 μl of dansyl chloride, and incubated at 45°C for 30 min to derive. Finally, 150 μl ammonia water was added and incubated at 45°C for 15 min. HPLC conditions and determination methods are described in 2.2.

Extraction of AFB1 from broad bean paste samples was performed according to the instruction of the producer supplied with the quantitative AFB1 test kits (CUSABIO, Wuhan, China).

### 2.10 The determination of volatile flavor compounds

The volatile flavor compounds in broad bean paste samples fermented for 30 days were determined and extracted using the solid‐phase microextraction (SPME) method according to Hu et al. (2021) and Kum et al. (2015). Three grams sample was taken and mixed with 10 μl cyclohexanone (Guangdong Guanghua Sci‐Tech) solution (10 μg/ml), which was an internal standard in a 20 ml headspace vial at 60°C water bath for 30 min. Inserting the SPME extraction head (75 μm CAR/PDMS) with adsorbed volatile compounds into the headspace vial, the extraction head was directly injected into the injector of gas chromatography (GC)‐mass spectrometry (GC–MS, Agilent Technologies Inc.) after 30 min. The sample was desorbed for 10 min at 250°C, and each sample was repeated three times. GC conditions: a DB‐5MS capillary column (30 m × 0.25 mm × 0.25 μm) was used with helium as carrier gas at a flow rate of 1.0 ml/min. The heating procedure was as follows: the column temperature was 35°C initially, maintained for 3 min, then increased to 50°C at 3°C/min, followed by a 6°C/min rise to 150°C, raised to 230°C at 10°C/min, then held at 230°C for 6 min. MS conditions: collection mode: full scan, collection mass range 40~350 m/z; ionization mode: electron bombardment (EI); emission energy: 70 EV; ion source temperature: 200°C; and interface temperature: 250°C. The flavor compounds were identified by searching the mass spectra and retention indices (RI) in the MS library of National Institute for Standards and Technology (NIST, Search v1.6) and Wiley (NY, 320 k compounds, v6.0).

### 2.11 Statistical analysis

All experiments were conducted in triplicate, and the statistical data were shown as the mean value ± standard deviation. SPSS 19.0 software package (SPSS Statistics) was used for one‐way analysis of variance (ANOVA) and Duncan's test (α = .05).

## 3 RESULTS

### 3.1 The LAB and yeast BA‐producing test in vitro

The BA‐producing ability of 16 LAB isolated from traditional Sichuan broad bean paste was determined to assess their safety. As shown in Table 1, putrescine, histamine, cadaverin, and β‐phenylethylamine were detected in tested strains. Most of the strains produced putrescine and histamine, indicating that these strains had ornithine and histidine decarboxylase activity. The strain with strong amine‐producing ability has the high activity of amino acid decarboxylase (Mah et al., 2019). Different kinds of microorganisms may produce different amino acid decarboxylases, which in turn, produce different biogenic amines. LAB were the main microorganisms that produced biogenic amines, among which the most common biogenic amines‐producing bacteria genus were *Enterococcus* (Jeon et al., 2018), *Lactobacillus* (Li et al., 2016), and *Lactococcus* (Ladero et al., 2011). Biogenic amines formation in broad bean paste was most likely related to the activity of LAB.

**TABLE 1:** Biogenic amines produced by LAB strains isolated from broad bean paste

Strain	Biogenic amines (mg/L)								
	Put	His	Trp	Cad	Phe	Tyr	Spm	Spd	Total
DPUL‐SC1	ND	0.69 ± 0.14^hi^,^1^	ND	ND	ND	ND	ND	ND	0.69 ± 0.10
DPUL‐SC2	1.92 ± 0.11^de^	2.47 ± 0.01^bc^	ND	ND	1.91 ± 0.04^a^	ND	ND	ND	6.30 ± 0.13
DPUL‐SC3	ND	0.19 ± 0.11^j^	ND	ND	ND	ND	ND	ND	0.19 ± 0.08
DPUL‐SC4	2.73 ± 0.06^b^	1.12 ± 0.04^efgh^	ND	ND	ND	ND	ND	ND	3.85 ± 0.05
DPUL‐J5	ND	0.97 ± 0.03^gh^	ND	ND	ND	ND	ND	ND	0.97 ± 0.02
DPUL‐SC6	2.19 ± 0.08^bcde^	2.49 ± 0.08^bc^	ND	ND	ND	ND	ND	ND	4.69 ± 0.12
DPUL‐SC7	2.58 ± 0.17^bc^	ND	ND	ND	ND	ND	ND	ND	2.58 ± 0.12
DPUL‐SC8	ND	1.05 ± 0.21^efgh^	ND	ND	ND	ND	ND	ND	1.05 ± 0.15
DPUL‐SC9	ND	0.23 ± 0.07^ij^	ND	ND	ND	ND	ND	ND	0.23 ± 0.05
DPUL‐SC10	2.07 ± 0.55^cde^	1.44 ± 0.14^efg^	ND	ND	ND	ND	ND	ND	3.51 ± 0.49
DPUL‐SC11	3.39 ± 0.16^a^	4.57 ± 0.23^a^	ND	ND	ND	ND	ND	ND	7.97 ± 0.26
DPUL‐SC12	ND	ND	ND	1.15 ± 0.20^a^	1.36 ± 0.18^a^	ND	ND	ND	2.51 ± 0.38
DPUL‐SC13	2.68 ± 0.61^bc^	2.01 ± 0.04^cd^	ND	ND	1.36 ± 0.18^a^	ND	ND	ND	6.14 ± 0.64
DPUL‐SC14	2.08 ± 0.06^cde^	0.96 ± 0.03^gh^	ND	ND	ND	ND	ND	ND	3.04 ± 0.06
DPUL‐SC15	2.51 ± 0.07^bcd^	1.90 ± 0.11^de^	ND	ND	ND	ND	ND	ND	4.41 ± 0.13
DPUL‐SC16	2.64 ± 0.08^bc^	2.60 ± 0.11^b^	ND	ND	ND	ND	ND	ND	5.25 ± 0.13

Abbreviations: cad, cadaverine; his, histamine; ND, not detected; phe, β‐phenylethylamie; Put, putrescine; spd, spermidine; spm, spermine; trp, tryptamine; tyr, tyramine.

^1^ Mean ± standard deviation. Mean values in the same column that are followed by different superscript letters are significantly different (*p* < .05).

Histamine and tyramine were the most dangerous BAs and are responsible for symptomatology known as “scombroid fish poisoning” and “cheese reaction,” respectively (Hungerford, 2010; McCabe‐Sellers et al., 2006). As mentioned by Yang et al. (2014), the maximum allowable concentration of histamine and tyramine range were 50–100 mg/kg and 100–800 mg/kg on food; beyond this range could cause migraines or severe poisoning symptoms. Strain DPUL‐SC11 had the strongest ability to produce biogenic amines, and the total biogenic amine content was 7.97 ± 0.26 mg/kg (putrescine 3.39 ± 0.16 mg/kg and histamine 4.57 ± 0.23 mg/kg). Strain with strong biogenic amine‐producing capacity was not suitable for starter culture in broad bean paste. Some strains had low biogenic amine‐producing capacity such as DPUL‐SC1, DPUL‐SC3, DPUL‐J5, and DPUL‐SC9. The strain with extremely low or no BAs was selected as a potential starter culture to improve the safety of broad bean paste.

As shown in Table 2, only four yeasts produced BAs, including putrescine, histamine, tryptamine, and β‐phenethylamine. The highest BAs content in the four yeasts was DPUY‐SC1 with a total BAs concentration of 9.02 ± 0.02 mg/kg and the content of β‐phenethylamine was 7.45 ± 0.01 mg/kg DPUY‐SC8 only produced putrescine with the concentration of 3.48 ± 0.02 mg/kg. Most of the yeast strains did not produce biogenic amines (including DPUY‐J5), such strains were suitable for the starter of broad bean paste, which reduced the risk factors (Torriani et al., 2010).

**TABLE 2:** Biogenic amines produced by yeast strains isolated from broad bean paste

Strain^1^	Biogenic amines (mg/L)								
	Put	His	Trp	Cad	Phe	Tyr	Spm	Spd	Total
DPUY‐SC1	ND	ND	1.57 ± 0.01^a,^ ^2^	ND	7.45 ± 0.01^a^	ND	ND	ND	9.02 ± 0.02
DPUY‐SC2	ND	ND	ND	ND	ND	ND	ND	ND	ND
DPUY‐SC3	3.17 ± 0.10^a^	2.80 ± 0.10^a^	ND	ND	ND	ND	ND	ND	5.98 ± 0.21
DPUY‐SC4	ND	ND	ND	ND	ND	ND	ND	ND	ND
DPUY‐J5	ND	ND	ND	ND	ND	ND	ND	ND	ND
DPUY‐SC6	ND	ND	ND	ND	ND	ND	ND	ND	ND
DPUY‐SC7	ND	ND	ND	ND	ND	ND	ND	ND	ND
DPUY‐SC8	ND	ND	ND	ND	ND	ND	ND	ND	ND
DPUY‐SC9	3.48 ± 0.02^a^	ND	ND	ND	ND	ND	ND	ND	3.48 ± 0.02
DPUY‐SC10	ND	ND	ND	ND	ND	ND	ND	ND	ND
DPUY‐SC11	ND	ND	ND	ND	ND	ND	ND	ND	ND
DPUY‐SC12	1.93 ± 0.01^b^	0.39 ± 0.20^b^	ND	ND	ND	ND	ND	ND	2.32 ± 0.22
DPUY‐SC13	ND	ND	ND	ND	ND	ND	ND	ND	ND
DPUY‐SC14	ND	ND	ND	ND	ND	ND	ND	ND	ND

Abbreviations: cad, cadaverine; his, histamine; ND, not detected; phe, β‐phenylethylamie; Put, putrescine; spd, spermidine; spm, spermine; trp, tryptamine; tyr, tyramine.

^1^ Yeast strains isolated from broad bean paste products.

^2^ Mean ± standard deviation. Mean values in the same column that are followed by different superscript letters are significantly different (*p* < .05).

### 3.2 Protease activity of LAB and yeast

During the fermentation process of broad bean paste, microorganisms will generate protease to catalyze the hydrolysis of polypeptides or proteins in the raw materials to generate various amino acids and increase the flavor components. The protease activity of the LAB and yeast strains in broad bean broth was measured as shown in Tables 3 and 4. The DPUL‐J5 showed the highest protease activity, which was 366.73 ± 9.00 U/L, and the DPUY‐J5 also showed the highest protease activity, which was 237.18 ± 10.93 U/L.

**TABLE 3:** Protease activity of lactic acid bacteria in Sichuan broad bean paste (U/L)

Strain	Protease activity (U/L)	Strain	Protease activity (U/L)
DPUL‐SC1	102.18 ± 7.71^f,^ ^1^	DPUL‐SC9	83.55 ± 8.36^gh^
DPUL‐SC2	83.55 ± 5.79^gh^	DPUL‐SC10	72.64 ± 1.93^hi^
DPUL‐SC3	95.36 ± 3.21^fg^	DPUL‐SC11	261.27 ± 0.00^b^
DPUL‐SC4	77.63 ± 9.00^hi^	DPUL‐SC12	94.00 ± 9.00^fg^
DPUL‐J5	366.73 ± 9.00^a^	DPUL‐SC13	106.27 ± 0.64^f^
DPUL‐SC6	64.00 ± 5.14^i^	DPUL‐SC14	93.09 ± 5.14^fg^
DPUL‐SC7	172.64 ± 9.64^d^	DPUL‐SC15	76.27 ± 4.50^hi^
DPUL‐SC8	130.82 ± 4.50 ^e^	DPUL‐SC16	224.91 ± 10.29^c^

^1^ Mean ± standard deviation. Mean values in the same column that are followed by different superscript letters are significantly different (*p* < .05).

**TABLE 4:** Protease activity of yeast in Sichuan broad bean paste (U/L)

Strain	Protease activity (U/L)	Strain	Protease activity (U/L)
DPUY‐SC1	217.64 ± 11.57^ab,^ ^1^	DPUY‐SC8	231.27 ± 6.43^ab^
DPUY‐SC2	47.18 ± 7.07^g^	DPUY‐SC9	127.64 ± 10.29^c^
DPUY‐SC3	212.18 ± 3.86^b^	DPUY‐SC10	29.46 ± 12.86^g^
DPUY‐SC4	103.09 ± 10.29^de^	DPUY‐SC11	96.73 ± 10.29 ^e^
DPUY‐J5	237.18 ± 10.93^a^	DPUY‐SC12	47.18 ± 3.22^g^
DPUY‐SC6	5.82 ± 3.87^h^	DPUY‐SC13	120.36 ± 15.43^cd^
DPUY‐SC7	68.55 ± 3.86^f^	DPUY‐SC14	103.09 ± 9.00^de^

^1^ Mean ± standard deviation. Mean values in the same column that are followed by different superscript letters are significantly different (*p <* .05).

### 3.3 Identification of the isolates

**FIGURE 2:**
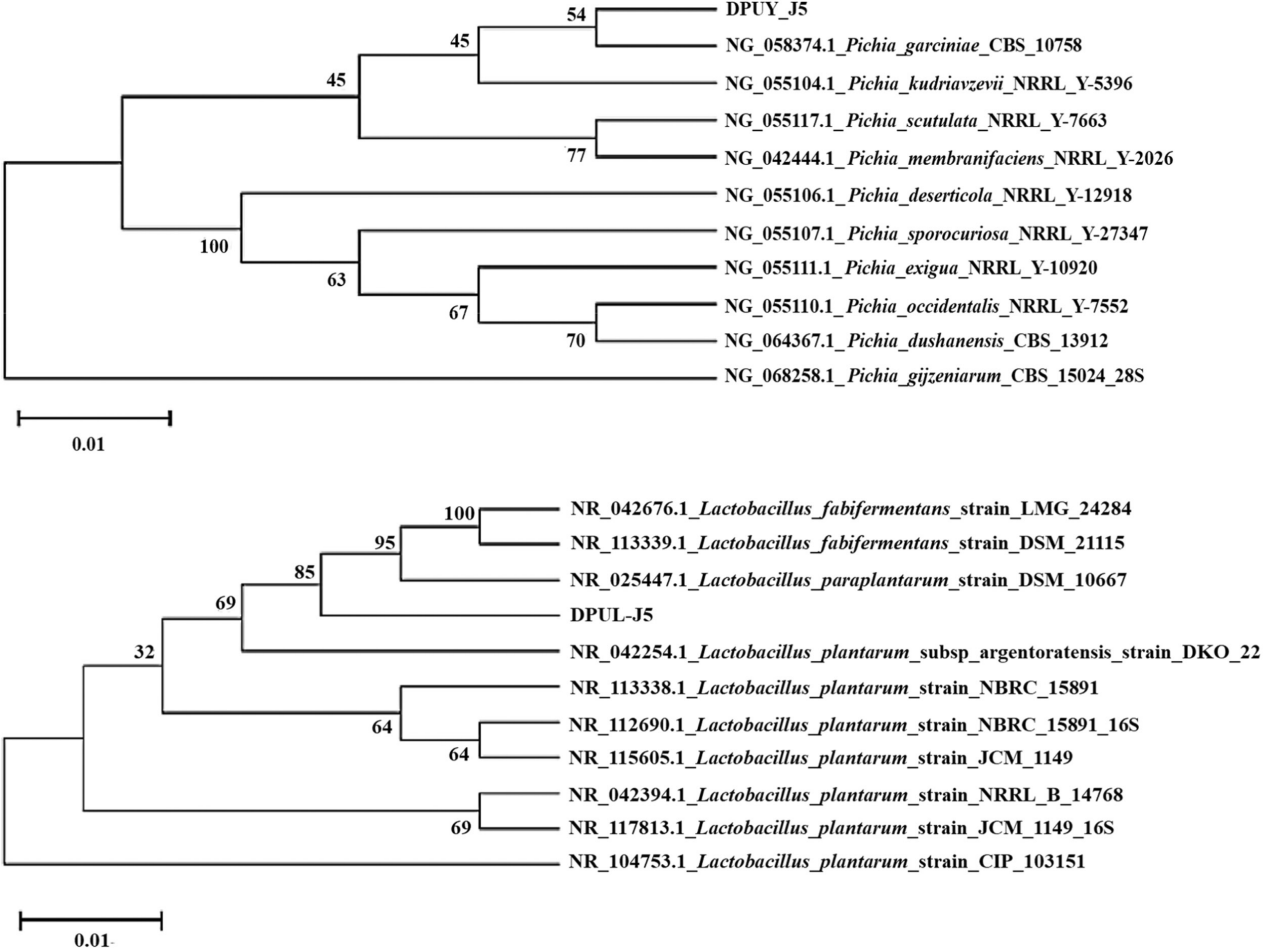
Phylogenetic tree of *Lactobacillus plantarum* DPUL‐J5 and *Pichia kudriavzevii* DPUY‐J5

According to the BA‐producing ability and protease activity results, LAB DPUL‐J5 and yeast DPUY‐J5 were selected for fermenting broad bean paste and were identified by 16S rRNA and 26S rRNA sequencing. The strain DPUL‐J5 was identified as *Lactobacillus plantarum* and named *L. plantarum* DPUL‐J5. The strain DPUY‐J5 was identified as *Pichia kudriavzevii* and named *P. kudriavzevii* DPUY‐J5. The phylogenetic tree of them is shown in Figure 2.

### 3.4 Antibiotic resistance

The antibiotic resistance properties of LAB strains about 10 common clinical antibiotics were evaluated according to the CLSI standards and the data were presented in Table 5. The results revealed that *L. plantarum* DPUL‐J5 was sensitive to clindamycin (CLDM), erythromycin (ERY), ampicillin (AMP), and chloramphenicol (CAP) antibiotics, and resistant to vancomycin (VAN) antibiotics. Ammor et al. (2007) reported that the lactobacilli strain was generally susceptible to antibiotics that inhibit the synthesis of proteins, such as chloramphenicol, erythromycin, clindamycin, and tetracycline, and more resistant to aminoglycosides (neomycin, kanamycin, streptomycin, and gentamicin). Most lactobacilli were resistant to vancomycin and carried resistance genes, however, the vancomycin resistance belonged to inherent resistance, which cannot be transferred to other strains (Varankovich et al., 2015). Meanwhile, *P. kudriavzevii* DPUY‐J5 was sensitive to clotrimazole (CLO), econazole (ECO), and itraconazole (ITR), and intermediate resistant to amphotericin (AMB), fluconazole (FLU), miconazole (MCZ), and ketoconazole (KCZ) (Table 6).

**TABLE 5:** Antibiotic susceptibility of *Lactobacillus plantarum* DPUL‐J5

Antibiotic discs	Content (μg)	*Lactobacillus plantarum* DPUL‐J5	
		Mean ± standard deviation	Antibiotic resistance^1^
KAN	30	15.88 ± 0.37^2^	I
CLDM	2	32.75 ± 0.36	S
ERY	15	38.76 ± 0.51	S
AMP	10	33.24 ± 0.79	S
LEV	5	16.85 ± 0.44	I
GEN	10	15.89 ± 2.32	I
Strep	10	15.75 ± 0.92	I
CAP	30	25.04 ± 0.83	S
TET	30	17.30 ± 1.55	I
VAN	30	–^3^	R

Abbreviations: AMP, ampicillin; CAP, chloramphenicol; CLDM, clindamycin; ERY, erythromycin; GEN, gentamicin; I, intermediate resistant; KAN, kanamycin; LEV, levofloxacin; R, resistant; S, sensitive; Strep, streptomycin; TET, tetracycline; VAN, vancomycin.

^1^ CLSI, Clinical and Laboratory Standards Institute. The inhibition zones are evaluated according to the standard values given by CLSI. Susceptible >20 mm, intermediate resistant 15–19 mm, and resistant ≤14 mm (CLSI, 2012).

^2^ Diameter of the inhibition zone including disc diameter.

^3^ Indicates no inhibition zone. Values are reported as means ± standard deviation of three separate replicates.

**TABLE 6:** Antibiotic susceptibility of *Pichia kudriavzevii* DPUY‐J5

Antibiotic discs	Content (μg)	*Pichia kudriavzevii* DPUY‐J5	
		Mean ± standard deviation	Antibiotic resistance^1^
AMB	30	10.06 ± 0.54^2^	I
FLU	2	10.80 ± 0.44	I
CLO	15	27.96 ± 2.51	S
MCZ	10	13.02 ± 0.03	I
KCZ	5	15.82 ± 1.11	I
ECO	10	20.43 ± 1.13	S
ITR	10	19.75 ± 0.50	S

Abbreviations: AMB, amphotericin; CLO, clotrimazole; ECO, econazole; FLU, fluconazole; I, Intermediate resistant; ITR, itraconazole; KCZ, ketoconazole; MCZ, miconazole; R, Resistant; S, Sensitive.

^1^ Diameter of the inhibition zone including disc diameter. Values are reported as means ± standard deviation of three separate replicates.

^2^ CLSI, Clinical and Laboratory Standards Institute. The inhibition zones are evaluated according to the standard values given by CLSI. Susceptible ≥20 mm, intermediate resistant 10–20 mm, resistant ≤10 mm.

### 3.5 Hemolytic activity

**FIGURE 3:**
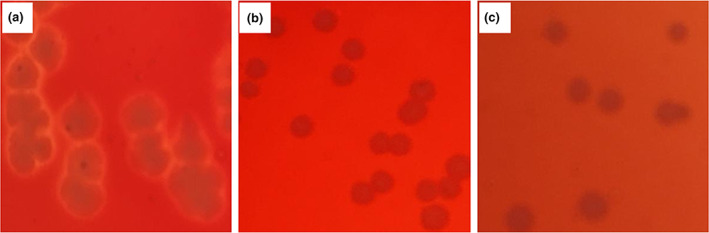
Hemolytic activity of *Lactobacillus plantarum* DPUL‐J5 and *Pichia kudriavzevii* DPUL‐J5. (a) α‐Hemolytic for *Listeria monocytogenes* (Positive control); (b) *Pichia kudriavzevii* DPUL‐J5; and (c) *Lactobacillus plantarum* DPUL‐J5

Hemolysis was the rupture and dissolution of red blood cells, which can be caused by a variety of physical and chemical factors and toxins. Certain hemolytic *Streptococcus* and *Bacillus perfringens* can cause septicemia (Nami et al., 2019). The hemolytic test showed that *Listeria monocytogenes* as positive control showed a clear hemolytic ring and had β‐hemolytic (Figure 3). The hemolytic activity test of *L. plantarum* DPUL‐J5 and *P. kudriavzevii* DPUY‐J5 showed negative results, indicating these strains were safe with no hemolytic activity.

### 3.6 Physicochemical analyses of the fermented broad bean paste

**FIGURE 4:**
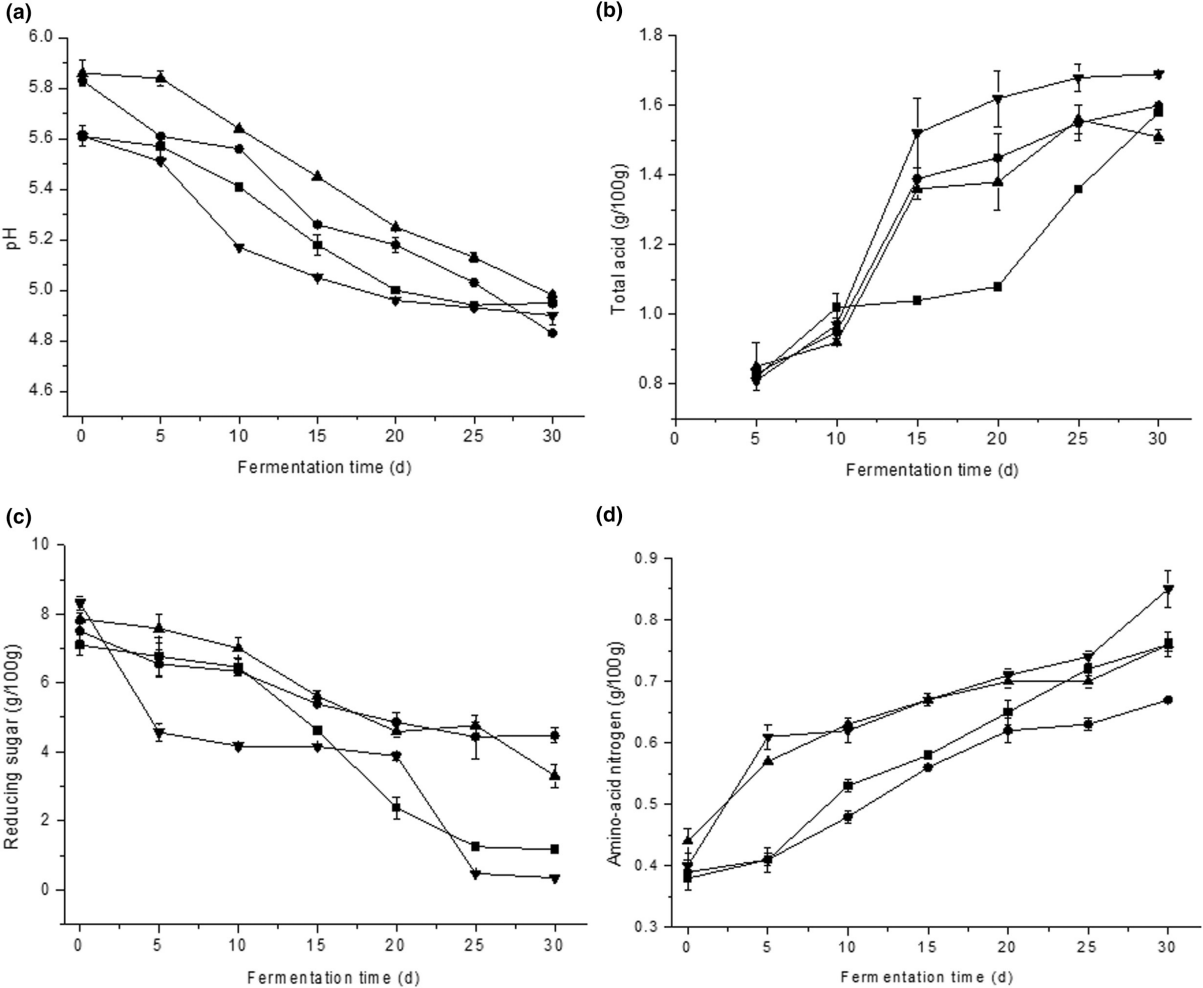
Physicochemical parameters in fermented broad bean paste with different fermation time. (a) pH; (b) total acid; (c) reducing sugar; and (d) amino‐acid nitrogen. (AB) Lines with solid square marks(■) representing inoculated with *Bacillus subtilis* DPUL‐J2; (ABL) lines with solid circle marks(●) representing inoculated with *Bacillus subtilis* DPUL‐J2 and *lactobacillus plantarum* DPUL‐J5; (ABP) lines with solid upward triangle marks(▲) representing inoculated with *Bacillus subtilis* DPUL‐J2 and *Pichia kudriavzevii* DPUL‐J5; (ABLP) lines with solid downward triangle marks(▼) representing coculture with *Bacillus subtilis* DPUL‐J2, *lactobacillus plantarum* DPUL‐J5, and *Pichia kudriavzevii* DPUY‐J5 (*p* < .05)

The physicochemical parameters, including pH values, the content of total acids, reducing sugar, and amino acid nitrogen in four kinds of broad bean pastes fermented with different strains, were determined (Figure 4). For the clarity of results, broad bean paste fermented by different strains was coded as sample AB, sample ABL, sample ABP, and sample ABLP, respectively.

The change in pH, total acid, reducing sugar, and amino nitrogen in broad bean paste were significant with the process of fermentation (*p* < .05). As shown in Figure 4a, the pH values of four kinds of broad bean paste samples fermented by different strains decreased, which was in accordance with the results of total acids concentration. The pH value of sample ABL (4.83) was the lowest while sample ABP (4.98) had the highest pH value. LAB produced lactic acid, which was the major metabolic end product of carbohydrates in food fermentation processes that inhibited the growth of competing microbes (Smid & Kleerebezem, 2014). Fermented foods with a pH value below 4–5 were usually considered safe, as most of the pathogens were unable to survive at this pH value (Padonou et al., 2010). The total acid concentration of sample ABL, sample ABP, and sample ABLP increased rapidly in the first 15 days and slowly in the later stage of fermentation, and the sample ABLP reached its highest at 1.69 g/100 g after 30 days of fermentation. The rapid increase in total acid content resulted from the mass reproduction of acid‐producing microorganisms in these samples. The variation trend of total acid in sample AB was different from other samples, which slowly increased in the first 20 days and then increased sharply. The total acid content of all samples did not exceed 2.00 g/100 g (Figure 4b). It was reported that LAB played important roles in the fermentation of Chinese soybean pastes, with the proliferation of LAB such as *Lactobacillus, Leuconostoc, Weissella*, and *Tetragenococcus halophilus* during soybean pastes fermentation, and acid contents of the samples increased gradually (Zhao et al., 2009).

Figure 4c showed the change in reducing sugar in broad bean paste samples. During the fermentation process of broad bean paste, the content of reducing sugars decreased continuously with the extension of the fermentation time. Many enzymes were produced by microorganisms in koji stage, among which amylase and glycosylase could hydrolyze starch grains in faba bean and produce a large amount of reducing sugar. Reducing sugar can provide a substantial carbon source for the growth of microorganisms. With the growth of microorganisms, reducing sugar was consumed continuously. After 30 days of fermentation, the reducing sugar content of sample ABLP was the lowest, so the utilization rate of reducing sugar was the highest. Therefore, during the fermentation stage, the change in reducing sugar content could help to understand the degree of hydrolysis of macromolecules in broad bean paste.

Amino acid nitrogen was usually considered as one of the main indices to represent broad bean paste quality. The protein in the broad bean was decomposed by microorganisms, resulting in the continuous increase in amino acid nitrogen. High amino acid nitrogen concentration might improve the quality of broad bean paste products. The amino acid nitrogen concentration was all increased since the start of fermentation and stabilized around 0.76 g/100 g, 0.67 g/100 g, 0.76 g/100 g, and 0.83 g/100 g for sample AB, ABL, ABP, and ABLP, respectively (Figure 4d). The results showed that the four samples had the same upward trend during the fermentation period. Consistent with the results of this study, Choi and Bajpai, (2010) also found that the total amino acid nitrogen was low at the beginning of fermentation, and tended to increase slightly as fermentation proceeded.

### 3.7 BA and AFB1 contents of broad bean paste

**FIGURE 5:**
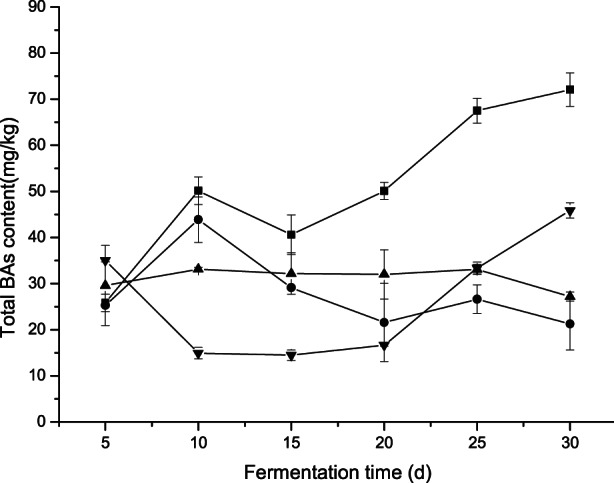
The contents of biogenic amines in fermented broad bean paste with different fermentation time. (AB) Lines with solid square marks(■) representing inoculated with *Bacillus subtilis* DPUL‐J2; (ABL) lines with solid circle marks(●) representing inoculated with *Bacillus subtilis* DPUL‐J2 and *lactobacillus plantarum* DPUL‐J5; (ABP) lines with solid upward triangle marks(▲) representing inoculated with *Bacillus subtilis* DPUL‐J2 and *Pichia kudriavzevii* DPUL‐J5; (ABLP) lines with solid downward triangle marks(▼) representing coculture with *Bacillus subtilis* DPUL‐J2, *lactobacillus plantarum* DPUL‐J5, and *Pichia kudriavzevii* DPUY‐J5 (*p* < .05)

As shown in Figure 5, the biogenic amine content of each group of samples was in a constantly changing process, and the biogenic amine content in all samples did not exceed the harmful level. Upper limits of biogenic amines in foods for human consumption have been suggested by several investigators as follows: histamine, 100 mg/kg; tyramine, 100–800 mg/kg; β‐phenylethylamine, 30 mg/kg; and total biogenic amines, 1000 mg/kg (Jeon et al., 2018). The BAs content of sample ABL on the 30th day was lower than sample AB, indicating that *Lactobacillus plantarum* DPUL‐J5 could degrade BAs or inhibit the BAs formation. Li et al. (2021) reported that *Lactobacillus plantarum* HM24 had a strong ability to degrade biogenic amines in the fermentation progress of soybean paste. In this study, BAs were detected at very low amounts in all tested samples of broad bean paste, which might be due to good raw material sources, the use of efficient starter cultures, adequate fermentation temperature and proper incubation, maintenance of proper hygiene, and proper storage (Shukla et al., 2014).

**FIGURE 6:**
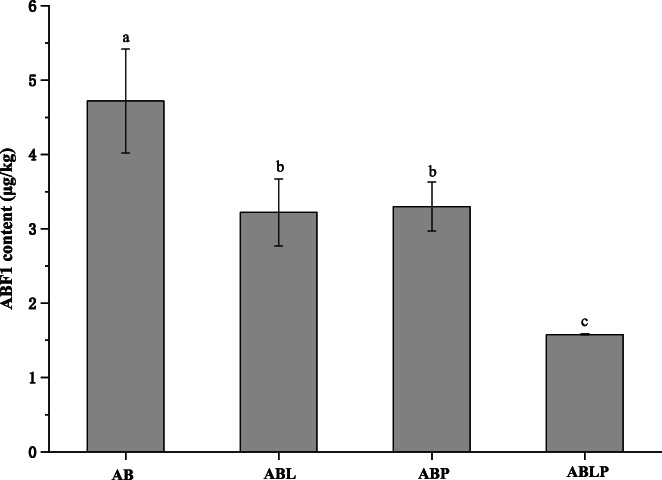
The concentration of AFB1 in fermented broad bean paste (*p* < .05)

The results of AFB1 levels in broad bean paste samples were determined as shown in Figure 6. The content of AFB1 in all samples did not exceed 5 μg/kg, meeting the “National standard for product of geographical indication‐Pixian douban” (GB/T 20560‐2006), which was not harmful to human health. Traditional broad bean paste was fermented in the open air, AFB1 can be produced when raw materials are improperly stored and contaminated by microorganisms. The safety of broad bean paste produced by traditional fermentation could not be guaranteed. In our study, the broad bean paste was fermented under good hygienic conditions and reduced by the contamination of mycotoxin‐producing fungi. Xia et al. (2017) screened a *Bacillus* strain, which exhibited ability to degrade aflatoxin B1 by 67.2%. Therefore, the starter culture was helpful to control the safety of broad bean paste fermentation when it did not produce AFB1 or degraded.

### 3.8 Volatile flavor compounds in broad bean paste

A total of 42 volatile flavor compounds were detected in broad bean paste samples after 30 days of fermentation, including eight alcohols, six aldehydes, four ketones, thirteen esters, five phenols, two alkenes, one alkane, one pyrazine, and two acids. In sample AB, the main volatile flavor compounds were alcohols, esters, and aldehydes, while few other flavor substances were detected (Table 7). However, in the sample ABL and ABP, more ketones, phenols, and other types of flavor compounds were detected. We found that higher concentrations of alcohols were detected in the sample ABP because alcohols were mainly produced by yeast fermentation. The phenylethyl alcohol concentration reached 2309.90 ± 43.98 ng/g (*p* < .05); it was reported that phenylethyl alcohol give the sauce a strong floral aroma (Jelen et al., 2013). The types of volatile flavor compounds in sample ABL and ABP were significantly lower than those in sample ABLP, and the increase in ester flavor compounds was particularly obvious in group ABLP, which was caused by the cofermentation of yeast and lactic acid bacteria (Xie et al., 2018). In the ABLP group, phenols contributed greatly to the overall flavor of broad bean paste, the concentration of 2‐methoxy‐4‐(1‐propenyl)‐phenol was noted to be 69.79 ± 8.75 ng/g (*p* < .05). Yin et al. (2022) found that 4‐ethyl‐guaiacol and 4‐ethyl‐phenol were the main phenolic compounds in chili paste, which were produced by the fermentation of *Aspergillus* and *Pichia bulbar*. The top three relative abundance of volatile flavor compounds in the fermentation broad bean paste were hexadecanoic acid–ethyl ester, linoleic acid–ethyl ester, and ethyl oleate. Hexadecanoic acid–ethyl ester and ethyl oleate had a wax‐like odor and a floral scent, and linoleic acid–ethyl ester had a slight astringency in sausage fermentation (Zhao et al., 2011). Thus, these compounds substantially contributed to the aroma of broad bean paste. Microorganisms played an important role in the fermentation of bean paste, LAB could provide the lactic acid, which together with yeast converted lactic acid and alcohols into esters, and aldehydes can be formed by the oxidation of alcohols and phenols (Yao et al., 2015). A large number of alcohols produced by yeast, or more aldehydes and ketones were reduced to alcohols during the fermentation (Zhao et al., 2021). Jeong et al. (2013) reported that the tastes, flavors, and qualities of kimchi were principally related to kimchi metabolites including organic acids, amino acids, mannitol, and carbohydrates, and the production of kimchi was influenced by the microbial community including LAB and yeasts present during fermentation. It is proved that the interaction of yeast and LAB can improve the types of volatile flavor compounds, but the quality of broad bean paste cannot be only judged by the types of volatile flavor compounds, and further quantitative identification of finished fermented products was needed.

**TABLE 7:** The volatile flavor compounds in fermented bean paste with different strains

Compounds	Retention time (min)	Concentration(ng/g)			
		Sample AB	Sample ABL	Sample ABP	Sample ABLP
Alcohols					
1‐Pentanol	4.18	ND	ND	171.18 ± 16.74	ND
2‐Furanmethanol	8.69	38.57 ± 0.18^1^	ND	ND	60.80 ± 6.33
(Z)‐2‐Hexen‐1‐ol	9.16	22.55 ± 1.56^c^	183.29 ± 5.44^a^	134.64 ± 19.88^b^	106.05 ± 25.86^b^
2,4‐Hexadien‐1‐ol	10.54	5.51 ± 0.13	ND	ND	96.50 ± 4.14
2‐ethyl‐2‐Hexen‐1‐ol	12.64	ND	ND	ND	358.81 ± 21.76
1‐Octen‐3‐ol	12.73	ND	ND	220.80 ± 35.24	ND
Linalool	16.22	108.14 ± 7.91^b^	98.57 ± 1.39^b^	129.69 ± 5.37^a^	92.19 ± 10.70^b^
Phenylethyl Alcohol	16.66	509.82 ± 81.48^d^	1697.93 ± 43.94^b^	2309.90 ± 43.98^a^	743.44 ± 17.78^c^
Aldehydes					
Benzaldehyde	12.00	291.93 ± 8.10^ab^	383.31 ± 54.42^a^	255.24 ± 37.87^b^	258.09 ± 37.71^b^
Benzeneacetaldehyde	14.60	702.42 ± 15.86^b^	886.74 ± 77.43^a^	647.11 ± 50.92^bc^	503.44 ± 58.91^c^
(E)‐2‐Octenal	15.11	ND	ND	ND	109.62 ± 18.32
Nonanal	16.34	96.33 ± 0.47^b^	76.71 ± 10.12^b^	133.57 ± 3.10^a^	96.09 ± 10.33^b^
Decanal	19.01	30.41 ± 8.13	35.99 ± 4.23	ND	39.84 ± 1.18
Benzenebutanal	20.32	54.35 ± 0.42^a^	ND	37.34 ± 4.87^b^	53.85 ± 5.79^a^
Ketones					
2‐Methyl‐cyclopentanone	10.41	23.25 ± 0.74	ND	ND	ND
5‐Methyl‐3‐heptanone	12.83	ND	ND	ND	549.72 ± 25.79
(E)‐6,10‐Dimethyl‐5,9‐undecadien‐2‐one	24.68	ND	16.06 ± 8.25	ND	ND
9‐Cedranone	25.80	ND	61.65 ± 11.19	ND	ND
Esters					
Octanoic acid, ethyl ester	18.80	ND	ND	30.11 ± 1.48	ND
Benzeneacetic acid‐ethyl ester	20.02	21.86 ± 0.62^c^	203.23 ± 18.76^ab^	169.79 ± 14.66^b^	239.89 ± 24.00^a^
Formic acid, 2‐phenylethyl ester	20.32	ND	ND	92.51 ± 6.64	ND
Allyl 2‐ethyl butyrate	22.64	ND	14.83 ± 2.70	ND	15.00 ± 1.31
Dodecanoic acid, ethyl ester	27.15	ND	ND	18.22 ± 0.09	ND
Tetradecanoic acid‐ethyl ester	29.80	84.13 ± 3.02^b^	81.56 ± 18.70^b^	251.46 ± 36.82^a^	94.17 ± 1.30^b^
Ethyl 13‐methyl‐tetradecanoate	30.61	150.04 ± 10.47^c^	289.50 ± 45.07^b^	490.23 ± 14.28^a^	133.83 ± 54.26^c^
Pentadecanoic acid‐ethyl ester	30.92	55.32 ± 1.87	ND	80.16 ± 12.46	ND
Ethyl 9‐hexadecenoate	31.76	ND	ND	53.30 ± 7.47	ND
Hexadecanoic acid‐ethyl ester	31.98	2046.56 ± 33.00^a^	1230.25 ± 81.01^b^	2003.28 ± 92.95^a^	943.10 ± 90.32^c^
Linoleic acid ethyl ester	33.70	50.87 ± 2.37^c^	767.12 ± 60.41^b^	2325.67 ± 124.22^a^	829.30 ± 90.08^b^
Ethyl Oleate	33.76	1077.93 ± 50.0^a^	756.34 ± 56.12^c^	1295.58 ± 97.69^a^	700.68 ± 29.37^c^
Octadecanoic acid‐ethyl ester	34.05	81.59 ± 2.24^a^	51.58 ± 12.81^a^	81.08 ± 26.91^a^	86.47 ± 6.66^a^
Phenols					
2‐Methoxy‐4‐vinylphenol	21.83	15.69 ± 0.66	ND	ND	ND
Eugenol	22.72	23.04 ± 1.73^a^	9.56 ± 0.88^b^	ND	14.77 ± 3.13^b^
2‐methoxy‐4‐(1‐propenyl)‐phenol	22.94	ND	56.92 ± 7.52	ND	69.79 ± 8.75
3‐Allyl‐6‐methoxyphenol	23.05	ND	ND	ND	1544.83 ± 195.09
4,6‐Di‐tert‐butyl‐m‐cresol	25.80	ND	58.51 ± 18.19	ND	ND
Olefins					
Guaiene	26.03	25.68 ± 0.71	ND	ND	ND
Hexadecane	27.20	ND	38.10 ± 3.15	ND	54.67 ± 3.11
Alkanes					
Tetradecane	23.46	ND	32.08 ± 2.78	ND	47.50 ± 14.67
Pyrazines					
Tetramethyl‐pyrazine	15.83	ND	ND	54.87 ± 6.63	185.27 ± 34.92
Acids					
Hexadecanoic acid	31.68	ND	139.24 ± 4.53^a^	119.47 ± 14.45^a^	61.13 ± 7.75^b^
Oleic acid	31.83	ND	107.89 ± 9.16	ND	43.87 ± 0.20

Abbreviation: ND, not detected.

^1^ Mean ± standard deviation. Mean values in the same column that are followed by different superscript letters are significantly different (*p <* .05).

Our results showed that the aroma compounds in the broad bean paste were influenced by the presence of *Lactobacillus plantarum* DPUL‐J5 and *Pichia kudriavzevii* DPUY‐J5 during broad bean paste fermentation. Alcohols, esters, and aldehydes were the major flavor components in the broad bean paste.

## 4 CONCLUSION

Strains of *Lactobacillus plantarum* DPUL‐J5 and *Pichia kudriavzevii* DPUY‐J5 were screened from traditional Sichuan broad bean paste which produces almost no biogenic amines, has high protease activity, and shows no resistance to antibiotics and hemolytic activity. The broad bean paste fermented by *Lactobacillus plantarum* DPUL‐J5 and *Pichia kudriavzevii* DPUY‐J5 was beneficial to the accumulation of total acid, amino acid nitrogen, and volatile flavor compounds while degrading harmful compound, such as biogenic amines. In a word, broad bean paste fermented with safe strains not only ensured the safety of the product but also improved the quality. Thus, this study provided reasonable suggestions for the standardization and safety of starter cultures in traditional broad bean paste production in Southwestern China.

## CONFLICT OF INTEREST

The authors state no potential conflicts of interest.

## Data Availability

All the data are all included in the manuscript.
